# Editorial: Long-Term Self-Tracking for Life-Long Health and Well-Being

**DOI:** 10.3389/fdgth.2021.827586

**Published:** 2021-12-24

**Authors:** Jochen Meyer, Cathal Gurrin, Blaine Price, Judy Kay, Ramesh Jain

**Affiliations:** ^1^OFFIS—Institute for Information Technology, Oldenburg, Germany; ^2^Insight Center for Data Analytics, Dublin City University, Dublin, Ireland; ^3^School of Computing and Communications, The Open University, Milton Keynes, United Kingdom; ^4^School of Computer Science, University of Sydney, Sydney, NSW, Australia; ^5^Department of Computer Science, University of California, Irvine, Irvine, CA, United States

**Keywords:** self-tracking, long-term, life-long health, well-being, health technology

Just a few years ago, it was only a small group of technology and data-loving nerds tracking their own health and behavior. Now and almost unnoticed, self-tracking has become a mass phenomenon. Activity trackers and sports watches, smartwatches and other wearables have become a common accessory on the wrists of a diverse range of people, young or old, fit or frail, cool or conservative. In this way, numerous people all around the world have been collecting personal health data under everyday conditions for years—a wealth of data that has received little attention to date.

Long-term self-tracking, collecting personal data over many years and decades, is a distinct flavor of general self-tracking (1). While there is no clear boundary to short-term tracking, there is a continuum of differences in aspects such as duration, goal and method of tracking, or approach to reflection (2). Particularly, while short-term tracking is more purposeful and driven by a user's specific need, long-term tracking is often more incidental and is done without a concrete reason out of a routine or for general and unclear interest.

With this article collection, we aim to shed light on some of the implications that these unique properties of long-term self-tracking have for concrete applications. Each of the papers in this collection explores a quite different perspective of the ways that people can make use of their growing collections of ever richer classes of long-term self-tracking health data.

Achieving a goal using self-tracking often requires a long term commitment to track but there has been little work done on how the mindset of the individual affects how long they will self-track before abandonment and how mindset affects reflection. Hanci et al. investigated this with 290 participants who voluntarily self-tracked their exercise. They found that those with fixed mindsets were more prone to negative self-talk which led to earlier abandonment. They note that the format of the feedback from most self-tracking systems comes in a one-size-fits-all format and suggest that designing tracking technologies with a mastery orientation for an open mindset may lead to a more meaningful and long-term engagement. Prompting a growth mindset and a more holistic appreciation of exercise can take the focus away from numbers and allow users to show more self-kindness.

Lupton addresses privacy concerns and willingness to share data among Australian long-term self- trackers. She interviewed a balanced, diverse group of 40 people who identified as tracking any aspect of their life (using any means). Her participants did not fit the “Quantified Self” stereotype nor did they post their data to social media: they either kept their data to themselves or shared it in very limited ways at limited times with close family, close friends/partners or health professionals. Reasons for sharing included wanting to inspire the recipient, for altruistic reasons, or to help the self-tracker achieve their goals. Most participants were not concerned about privacy breaches or their data leaking, which reflects their high level of faith in the device manufacturers and cloud providers to protect their data and not allow de-anonymization–a faith that, as other studies have shown, is probably misplaced.

Li et al. tackle the challenge of how to make sense of long-term self-tracking data to support accurate self-awareness for the individual. They point out that most of the prior research studies on self-awareness use data that has been gathered in controlled or laboratory settings. This paper proposes that lifelog data, in its many forms, can offer new opportunities for understanding self-awareness, which has potential for enhancing the development and well-being of the individual. Specifically they focus on four important topics of sleep quality prediction, personality detection, mood detection/prediction, and depression detection. Their experiments on real-world lifelog datasets show promising results across all four topic domains.

Last but not least, Faust et al. present an application making use of self-tracking data by investigating how severe negative life events such as the death of a loved one are reflected in fitness tracker data. They equipped a group of more than 750 professionals with a smart watch for 1 year and analyzed the data of those 45 who reported a significant negative life event, enabling them to demonstrate physiological responses to the negative life event. This is an excellent example of how incidental long-term self-tracking data can retrospectively be repurposed for an application that was not known at the time of collecting the data.

By investigating self-tracking behavior, data analysis, and potential applications as well as potential privacy risks, the articles in this collection address important aspects of long-term self-tracking. They contribute to the growing body of work about this evolving topic. As the devices for self-tracking of health measures continue to become cheaper and more powerful, they are creating new ways for people to gain valuable insights from their long term data.

## Author Contributions

All authors listed have made a substantial, direct, and intellectual contribution to the work and approved it for publication.

## Conflict of Interest

RJ and CG hold some patents and are co-founders of start-ups. The remaining authors declare that the research was conducted in the absence of any commercial or financial relationships that could be construed as a potential conflict of interest.

## Publisher's Note

All claims expressed in this article are solely those of the authors and do not necessarily represent those of their affiliated organizations, or those of the publisher, the editors and the reviewers. Any product that may be evaluated in this article, or claim that may be made by its manufacturer, is not guaranteed or endorsed by the publisher.
